# Elucidating Therapeutic and Biological Potential of *Berberis baluchistanica* Ahrendt Bark, Leaf, and Root Extracts

**DOI:** 10.3389/fmicb.2022.823673

**Published:** 2022-03-09

**Authors:** Zareen Gul, Ali Akbar, Saadullah Khan Leghari

**Affiliations:** ^1^Department of Botany, University of Balochistan, Quetta, Pakistan; ^2^Department of Microbiology, University of Balochistan, Quetta, Pakistan

**Keywords:** ethanolic extracts, antimicrobial, anti-inflammatory, anti-leishmanial, anticancer, hemolysis inhibition

## Abstract

*Berberis baluchistanica* Ahrendt is a medicinal plant known to have potential for the treatment of various diseases. In the present study, the ethanolic extracts of the bark, leaves, and roots of *B. baluchistanica* plant were evaluated for *in vitro* antimicrobial, anti-leishmanial, anticancer, and anti-inflammatory activities. The antibacterial and antifungal activities were determined by agar mix and agar well diffusion method. All extracts showed potential activity against the target bacteria (*Bacillus subtilis*, *Bacillus licheniformis*, *Escherichia coli*, *Klebsiella pneumoniae*, *Pseudomonas aeruginosa*, *Rhodococcus erythropolis*, *Salmonella typhi*, and *Staphylococcus aureus*) and fungal strains (*Aspergillus flavus*, *Aspergillus niger*, and *Mucor mucedo*). *S. aureus* proved to be the most sensitive strain for each extract, with a maximum zone of inhibition for bark at 23 ± 0.12 mm, for leaves at 22 ± 0.36 mm, and for root extracts at 20.21 ± 0.06 mm). The minimum inhibitory concentration values of *B. baluchistanica* bark, leaves, and roots for different target bacterial strains ranged from 1.56 to 25 mg ml^–1^, and the minimum bactericidal concentrations were in the range of 3.12 to 25 mg ml^–1^, respectively. The root extract possessed potent antifungal activity against *A. flavus* with 83% of growth inhibition, *A. niger* with 80%, and *M. mucedo* with 73%. The bark extract was found active against *M. mucedo* with 86% of inhibition, followed by 70% against *A. flavus* and 60% against *A. niger.* The leaf extract showed a significant response by 83% inhibition against *M. mucedo*, followed by *A. flavus* and *A. niger* with 73 and 72% inhibition, respectively. In an anti-leishmanial bioassay, the inhibitory concentration (IC_50_) was observed for each extract against *Leishmania major*. The bark showed good activity (IC_50_ = 4.95 ± 0.36 mg/ml), followed by the roots (IC_50_ = 7.07 ± 0.18 mg/ml) and the leaves (IC_50_ = 8.25 ± 0.29 mg/ml). An evaluation of anticancer activity was done by using MTT cell assay against HeLa cell line. Upon comparing the values of each extract to the standard, it was revealed that the ethanolic bark extract showed the highest anticancer activity with IC_50_ = (12 ± 0.15 μg/ml), followed by the roots (14 ± 0.15 μg/ml) and the leaves (17 ± 0.21 μg/ml), respectively. The anti-inflammatory assay was undertaken by the inhibition of albumin denaturation activity, proteinase inhibitory activity, and heat-induced hemolysis activity. The IC_50_ value for protein denaturation of the bark was IC_50_ = 0.64 ± 0.25 mg/ml, followed by the roots (0.67 ± 0.21 mg/ml) and the leaves (0.73 ± 0.13 mg/ml). The proteinase inhibitory activity of the bark extract was IC_50_ = 0.55 ± 0.12 mg/ml, followed by the leaves (0.62 ± 0.23 mg/ml) and the roots (0.69 ± 0.15 mg/ml), respectively. For heat-induced hemolysis assay, the bark showed the lowest IC_50_ value (0.48 ± 0.15 mg/ml) compared to the leaves (0.52 ± 0.35 mg/ml) and the roots (0.58 ± 0.05 mg/ml) of the plant. All analyzed parts of the *B. baluchistanica* plant showed significant biological activities which make the plant medicinally important and a good candidate for the isolation of antimicrobial, inflammatory, and anticancer compounds. Further studies may lead us to determine the active compounds responsible for the biological activities of the plant extracts.

## Introduction

The use of medicinal plants as an alternative therapy for the prevention and treatment of various infectious diseases is an ancient practice (Tareen et al., 2016). Long before mankind, the subsistence of microbes was discovered; the idea was well accepted that some medicinal plants that contain active components belonging to different phytochemical groups had antimicrobial potential (Rios and Recio, 2005). The curative properties of various medicinal plants have been known to treat human and animal diseases. It is estimated by the World Health Organization (WHO) that about 80% of the world’s population still rely on traditional medicine for their primary healthcare needs due to their easy availability (Dos Santos et al., 2021). Medicinal plants with bioactive components having antibacterial, antifungal, and antioxidant properties are used for the treatment of various infections. These natural antimicrobial agents have great medicinal potentials because of the increasing resistance to the usual antibiotics and drugs (Pervez et al., 2019).

Antibiotics are one of the most essential weapons to be used against various infections and have greatly enhanced the quality of human life. However, in the last decades, these health advantages had lost their effectiveness not only because their many contraindications but also due to the rapid increase of multidrug resistance. Medicinal plants might represent an alternative treatment in the treatment of infectious diseases (Sailaja, 2014). They can also be a possible source for new potent antibiotic molecules to which pathogenic strains are not resistant. It is necessary to explore new drugs, for the healthcare system in developing countries, which could be cheaper and affordable and with a lower chance of resistance produced by common pathogens (Banik et al., 2014). Thus, the use of traditional therapies has prompted scientists to analyze the biological potentials of medicinal plants against various infection agents and to prove their effectiveness for their safe traditional uses.

Considering the vast potentiality of plants as a source for antimicrobial drugs, a systematic analysis was carried out to screen the local flora *Berberis baluchistanica* Ahrendt, locally known as Zralag in Pashto, Zarch in Brahvi, and Korae in Balochi, for different biological activities. It is endemic to Balochistan and belongs to family Berberidaceae. The plant is distributed in Harboi, Kalat, and Zarghun areas of Quetta and Ziarat (Uddin et al., 2021). This medicinal plant is valued for its bark and roots, considered as non-toxic and consumed in raw form either as powder or a decoction. As it contains berberine, the plant is used for the treatment of various diseases, like cough, fever, internal injury, eye disease, kidney stone removal, wound healing, rheumatism, and other infections of human beings and livestock (Baloch et al., 2013). Recently, various secondary metabolites, like berberisinol, berberine, 8-oxoberberine, oleanolic acid, palmatine, gallic acid, phenols, carotenoids, and vitamins, were isolated and found with remarkable antioxidant, anti-leishmanial, anti-diabetic, antibacterial, and antifungal potential (Pervez et al., 2019).

The *in vitro* antibacterial, antifungal, anti-inflammatory, anti-leishmanial, anticancer, and detailed biological potential of the bark, leaf, and root ethanolic extracts of *B. baluchistanica* has not been reported to date. Therefore, the plant was tested for the development of an alternative drug line, and the present study was carried out to evaluate the antibacterial, antifungal, anti-inflammatory, anti-leishmanial, and anticancer effects of the bark, leaf, and root extracts of the plant against different microorganisms by using an *in vitro* assay method.

## Materials and Methods

### Plant Collection

*Berberis baluchistanica* plant was collected from the Ziarat region of Balochistan and identified by Dr. Saadullah Khan Leghari, professor at the Department of Botany, University of Balochistan Quetta. The voucher specimens were prepared, and the plant was deposited in the Herbarium, Department of Botany, University of Balochistan Quetta, Pakistan.

### Sample Preparation

The bark, leaves, and roots of the plant were separated, washed off with distilled water in order to remove any kind of dust and contamination, and dried under the shade for 3 to 4 consecutive weeks at room temperature and under controlled humidity. The dried bark, leaves, and roots were separately ground into fine powder using an electrical grinder and stored in desiccators for further analysis.

### Maceration Extraction of Bioactive Compounds

In maceration extraction, 200 g of fine grounded powder was used for extraction using 2 L ethanol as solvent with 1:10 ratio, following standard procedures described by Akbar et al. (2019a). The process was conducted in a dark room to avoid light exposure. The flask was occasionally shaken with specific intervals. The ethanolic mixture was filtered with Whatman filter paper no. 1. Each extract was dried with the help of a rotary evaporator and used for further analysis.

### Biological Analysis

The following biological activities were performed on the bark, leaves, and roots of *B. baluchistanica.*

#### Antibacterial Activity

The antibacterial activity of *B. baluchistanica* bark, leaf, and root extracts was determined by using agar well diffusion method (Akbar et al., 2019a). Freshly prepared sterilized culture media were inoculated with the target bacterial strains (*Bacillus subtilis*, *Bacillus licheniformis*, *Escherichia coli*, *Klebsiella pneumoniae*, *Pseudomonas aeruginosa*, *Rhodococcus erythropolis*, *Salmonella typhi*, and *Staphylococcus aureus*) and incubated at 37°C for 24 h. The target bacterial cultures were spread over the surface of sterilized Muller Hinton Agar (Oxoid, UK) plates with the help of a sterilized cotton swab for the determination of antibacterial activity. Wells were made through 6-mm sterilized cork borers in agar plates, and extracts were added into the agar wells. Dimethyl sulfoxide (DMSO) was used as negative control, and the antimicrobial drug doxycycline was used as a positive control. The plates were incubated at 37°C for 24 h. The results were interpreted by measuring the diameter of the clear zone around the wells in millimeter (mm).

#### Minimum Inhibitory and Minimum Bactericidal Concentration

The minimum inhibitory concentration (MIC) and minimum bacterial concentration (MBC) of each extract were determined by the method described by Sadiq et al. (2017) with minor modifications. Briefly, 50 mg/ml stock solution of each extract was prepared in DMSO. Twofold serial dilutions of each extract were prepared in sterile nutrient broth as per the manufacturer’s instruction to obtain the concentrations of 50, 25, 12.5, 6.25, 3.12, and 1.56 mg/ml. Each bacterial inoculum of approximately 10^5^–10^6^ colony-forming units (CFU)/ml was introduced in each concentration of the extract and incubated at 37°C for 24 h in an incubator. The test tubes containing the extract-free nutrient broth with bacterial inoculums were used as the positive control, and the test tubes containing the extract and nutrient broth without inoculums were used as the negative control. The lowest concentration which showed no visible growth after 24 h of incubation period was considered as the MIC. The MBC determined by standard plate count method for the target bacteria by subculturing 1 ml from each dilution that had no eye-visible growth was inoculated on the surface of freshly prepared nutrient agar plates with the help of a sterile spreader and incubated at 37°C for 24 h. Each experiment was conducted in triplicates. The minimum concentration that had no visible growth on the agar plates after 24 h of incubation was measured as the MBC.

#### Kill Time Assay

Kill time study was used to calculate the bactericidal effects of the bark, leaves, and roots of *B. baluchistanica* following Akbar and Anal (2014) with slight modifications. Stock solutions (50 mg/ml) of each extract were used to prepare different dilutions by mixing with nutrient broth in clean and dried test tubes and sterilized under pressure at 121°C for 15 min in an autoclave. The kill time curves were established by inoculating the target bacterial strains *B. subtilis*, *B. licheniformis*, *E. coli*, *K. pneumoniae*, *P. aeruginosa*, *R. erythropolis*, *S. typhi*, and *S. aureus* at approximately 10^6^CFU/ml in the broth containing each extract. Nutrient broth containing test bacteria without extract was used as the positive control. The growth and turbidity pattern of the inoculated test bacterial strains were observed at specific time intervals (0, 2, 6, 16, and 24 h) with the help of a spectrophotometer (T60 UV–Vis, PG UK) at 625 nm. The growth of the test bacterial species was also confirmed with the help of a standard plate count technique.

#### Antifungal Activity

The antifungal activities of the extract of *B. baluchistanica* was determined by agar well diffusion method following Akbar et al. (2019a). Briefly, 2 ml of each extract was dissolved in DMSO and transferred to freshly prepared 35-ml potato dextrose agar (Oxoid UK), shaken properly, and poured into petri plates. After solidification, 6-mm-diameter wells were made into the agar by a cork borer, and the fresh cultures of test fungi (*A. flavus*, *A. niger*, and *M. mucedo*) were poured into different wells and incubated at 37°C for 72 h. Media having organisms without extract and media alone were used as positive and negative controls, respectively. The antifungal drug fluconazole was used as the reference. The results of the growth inhibition of the target fungal species were recorded after 5 days of incubation. The growth inhibition of the target fungi was determined by measuring the linear growth in millimeter (mm) with reference to the negative control. The final results were calculated by the following equation:


%inhibition=100-lineargrowthintest(mm)/linear⁢growth⁢in⁢control⁢(mm)×100

#### *In vitro* Anti*-*leishmanial Susceptibility Assay

##### Parasite Culture

The promastigotes of *Leishmania major* were isolated from the previously isolated and preserved culture stored in the Food Microbiology and Bioprocess Laboratory, Department of Microbiology, University of Balochistan Quetta. The promastigotes were grown in NNN biphasic medium with penicillin and streptomycin.

The anti-leishmanial activity assay was done by the method previously described (Shaheen et al., 2020) but with minor modifications. The log phase of promastigotes at 1 × 10^6^ cells/ml was used for the current assay. The ethanolic extracts of the bark, leaves, and roots of the medicinal plant *B. baluchistanica* were examined against *L. major* (promastigotes) in culture by means of a 96-well plate. The promastigotes of *L. major* were grown in NNN biphasic medium. Briefly, a stock solution (1 mg/ml) of the test sample was prepared in DMSO. A twofold serial dilution of each sample was carried out, and the activity was performed at different concentrations (1, 0.5, 0.25, 0.125, and 0.0625 mg/ml). Then, 10 μl of each plant dilution and 50 μl of the promastigote log-phase culture was dispensed to each well of a 96-well plate. Glucantime was used as a standard drug. Media with organism and DMSO were maintained as positive and negative controls, respectively. The titer plate was incubated for 72 h at 37°C. The assay was performed in triplicate. After incubation, 1 ml of DMSO was added to each well, and the percent mortality of the test and standard drug was confirmed by using 20 μl (5 mg/ml in phosphate buffer, pH 7.2) of NBT solution. The absorbance was measured at 630 nm by using Microplate Reader (RT- 6000), and the IC_50_ values of each extract with anti-leishmanial activities were calculated by the linear regression method. The percent of cell viability was calculated by using the following formula:


$$\%\,\mathrm{cell}\,\mathrm{viability}={\text{A}}_{630}\,\mathrm{of}\,\mathrm{test}\,\mathrm{sample}/{\text{A}}_{630}\,\mathrm{of}\,\mathrm{control}\times 100\%\text{inhibition}=100-\%\text{viability}$$

#### MTT Cell Assay

##### Cell Culture

HeLa cell line (cervical cancer carcinoma) was cultured in a humidified atmosphere with 5% CO_2_ in minimal essential medium supplemented with 10% FBS, 100 units/ml penicillin, and 100 μg/ml streptomycin at 37°C.

The cytotoxic effect of the ethanolic extracts of the bark, leaves, and roots against HeLa (cervical cancer carcinoma) was determined by a rapid colorimetric assay using MTT 3-(4, 5- dimethylthiazol-2-yl)-2, 5-diphenyltetrazolium bromide (Javed et al., 2021). Briefly, 180 μl of cell suspension (1 × 10^5^ cell/ml) was seeded in 96-well plates and incubated at 37°C at 5% CO_2_ condition, treated with 100 μg/ml of each extract, and incubated for 48 h. The cell viability was evaluated by dissolving MTT in phosphate-buffered saline (PBS, pH 7.2), and 20 μl (5 mg/ml in phosphate buffer) of 0.5% 3-(4, 5-dimethylthiazol-2-yl)-2, 5-diphenyltetrazolium bromide (MTT solution) was added and incubated for 3 h. After incubation, the supernatant in each well was carefully removed, and 1 ml of DMSO was added to each well. The amount of formazan formed was determined by measuring the absorbance at 570 nm with a UV spectrophotometer. Doxorubicin (100 μg/ml) was used as the standard and DMSO was used as the negative control, respectively. A standard curve (absorbance against the number of cells) was plotted, and measurements were performed to calculate the concentration required for 50% inhibition (IC_50_). The percent of cell viability was calculated by using the following formula:


$$\%\mathrm{cell}\mathrm{viability}={\text{A}}_{570}\mathrm{of}\mathrm{treated}\mathrm{cells}/{\text{A}}_{570}\mathrm{of}\mathrm{control}\times 100$$

#### Assessment of *in vitro* Anti-inflammatory Activity

##### Protein Denaturation Assay

The anti-inflammatory activity of *B. baluchistanica* bark, leaf, and root extracts was carried out by inhibition of protein denaturation assay according to the method of Yesmin et al. (2020) with slight modifications. The reaction mixture (5 ml) consisted of 0.2 ml of 1% bovine albumin, 3.8 ml of PBS (pH 6.4), and 1 ml of each extract (1 mg/ml), was mixed, and was incubated in a water bath (37°C) for 15 min. Then, the reaction mixture was heated at 70°C for 5 min. After cooling, the absorbance was measured at 660 nm using a spectrophotometer (T60 UV-Vis. PG UK). Phosphate buffer solution was used as control. The percentage inhibition of protein denaturation was calculated by using the following formula:


%inhibitionofproteindenaturation=100×(1-A2/A1)

where A1 = absorption of the control sample, and A2 = absorption of the test sample.

#### Proteinase Inhibitory Activity

To examine the *in vitro* anti-inflammatory action, proteinase inhibitory activity of the bark, leaf, and root extracts was performed according to the method of Gunathilake et al. (2018). The reaction solution (2 ml) consisted of 0.06 mg trypsin, 1 ml of 20 mM Tris–HCl buffer (pH 7.4), and 1 ml of each extract dilution sample. The mixture was incubated (37°C for 5 min), and then 1 ml of 0.8% (w/v) casein was added, followed by further incubation for 20 min. At the end of incubation, 2 ml of 70% perchloric acid was added to conclude the reaction. The mixture was centrifuged, and the absorbance of the supernatant was measured at 210 nm. Phosphate buffer solution was used as the control, and aspirin was used as the standard drug for the experiment. The percentage inhibition was calculated by using the following formula:


%⁢proteinase⁢inhibition=(Abs⁢control-Abs⁢sample)×100/Abs⁢control

#### Heat-Induced Hemolysis Assay

##### Preparation of Erythrocyte Suspension

Erythrocyte suspension was prepared according to the method described in Yesmin et al. (2020) with some modifications. Whole human blood was collected from a healthy human. Sodium oxalate was used to prevent blood clotting. For supernatant removal, the blood was centrifuged at 2,500 rpm for 5 min. The mixture was washed with an equal volume of normal saline (0.9% NaCl), and centrifugation was done at 2,500 rpm for 5 min. The process was done three times, and the blood volume was measured and reconstituted as 10% (v/v) suspension with isotonic buffer solution (10 mM sodium phosphate buffer, pH 7.4). The composition of the buffer solution (g/L) used was NaH_2_PO_4_⋅2H_2_O (0.2 g), Na_2_HPO_4_ (1.15 g), and NaCl (9.0 g).

The heat-induced hemolysis activity was carried out as described by Gunathilake et al. (2018) with some modifications. Briefly, 0.05 ml of blood cell suspension and 0.05 ml of each extract were mixed with 2.95 ml phosphate buffer (pH 7.4). The mixture was incubated at 54°C for 20 min in water bath. After the incubation, the mixture was centrifuged (2,500 rpm for 3 min), and the absorbance of the supernatant was measured at 540 nm using a spectrophotometer (T60 UV–vis. PG UK). Phosphate buffer solution was used as the control, and aspirin was used as the standard drug for the experiment. The percent inhibition of hemolysis was calculated using the following equation:


Percentage⁢inhibition⁢of⁢hemolysis=(Abs⁢control-Abs⁢sample)×100/Abs⁢control

where A1 = absorption of the control and A2 = absorption of the test sample.

### Statistical Analysis

The results of all the measured data in each experiment were expressed as average ± standard deviations (SD). The magnitude of the means, standard curve, and standard deviations were calculated by using MS Excel 2010 Software. The inhibitory concentrations (IC_50_) were measured by the linear regression method. Significant group difference (*p* < 0.05) between means was determined by using SPSS software.

## Results

### Antibacterial Activity

**FIGURE 1:**
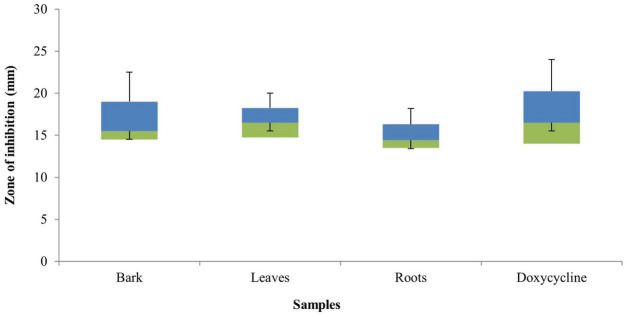
Box plot graph of the antibacterial activity of *Berberis baluchistanica* bark, leaf, and root extracts against various bacterial strains. Bars represent the standard deviation of the mean.

The bark, leaf, and root extracts of the *B. baluchistanica* plant was tested for its antibacterial properties against different bacterial strains. The diameter of the inhibition zones of the bark extract against *B. subtilis*, *B. licheniformis*, *E. coli*, *K. pneumonia*, *P. aeruginosa*, *R. erythropolis*, *S. typhi*, and *S. aureus* was 15 ± 0.23, 18 ± 0.54, 22 ± 0.51, 13 ± 1.16, 12 ± 1.13, 16 ± 0.12, 15 ± 0.74, and 23 ± 0.36 mm, respectively. The leaf extract showed a relatively higher activity, and the zone of inhibition against *B. subtilis* was 16 ± 1.01 mm, *B. licheniformis* 17 ± 1.02 mm, *E. coli* 19 ± 0.47 mm, *K. pneumonia* 16 ± 0.61 mm, *P. aeruginosa* 11 ± 0.15 mm, *R. erythropolis* 18 ± 1.12 mm, *S. typhi* 10 ± 0.53 mm, and *S. aureus* 22 ± 0.12 mm. The calculated results for the root extracts were 12 ± 0.41, 14 ± 0.32, 23.14 ± 1.16, 7 ± 0.01, 14 ± 0.41, 15 ± 0.24, 14 ± 1.06, and 20.21 ± 0.06 mm against *B. subtilis*, *B. licheniformis*, *E. coli*, *K. pneumonia*, *P. aeruginosa*, *R. erythropolis*, *S. typhi*, and *S. aureus* (Table 1). All the extracts were found active against the selected target bacteria, except *K*. *pneumonia* that was resistant to root extracts (Figure 1).

**TABLE 1:** Antibacterial activity of different parts of *Berberis baluchistanica* with inhibitory zones in mm as mean ± standard deviation.

	Diameter of zone of inhibition (mm ± SD) against pathogens							
	
Samples	*Bacillus subtilis*	*Bacillus licheniformis*	*Escherichia coli*	*Klebsiella pneumoniae*	*Pseudomonas aeruginosa*	*Rhodococcus erythropolis*	*Salmonella typhi*	*Staphylococcus aureus*
Bark	15 ± 0.23	18 ± 0.54	22 ± 0.51	13 ± 1.16	12 ± 1.13	16 ± 0.12	15 ± 0.74	23 ± 0.36
Leaves	16 ± 1.01	17 ± 1.02	19 ± 0.47	16 ± 0.61	11 ± 0.15	18 ± 1.12	10 ± 0.53	22 ± 0.12
Roots	12 ± 0.41	14 ± 0.32	23.14 ± 1.16	7 ± 0.01	14 ± 0.41	15 ± 0.24	14 ± 1.06	20.2 ± 0.1
Doxycycline	12 ± 0.03	20 ± 0.14	23 ± 0.13	21 ± 0.32	10 ± 0.05	18 ± 0.1	15 ± 0.91	14 ± 0.02

*mm, millimeter; SD, standard deviation. Doxycycline = control.*

### Minimum Inhibitory and Minimum Bactericidal Concentration

**FIGURE 2:**
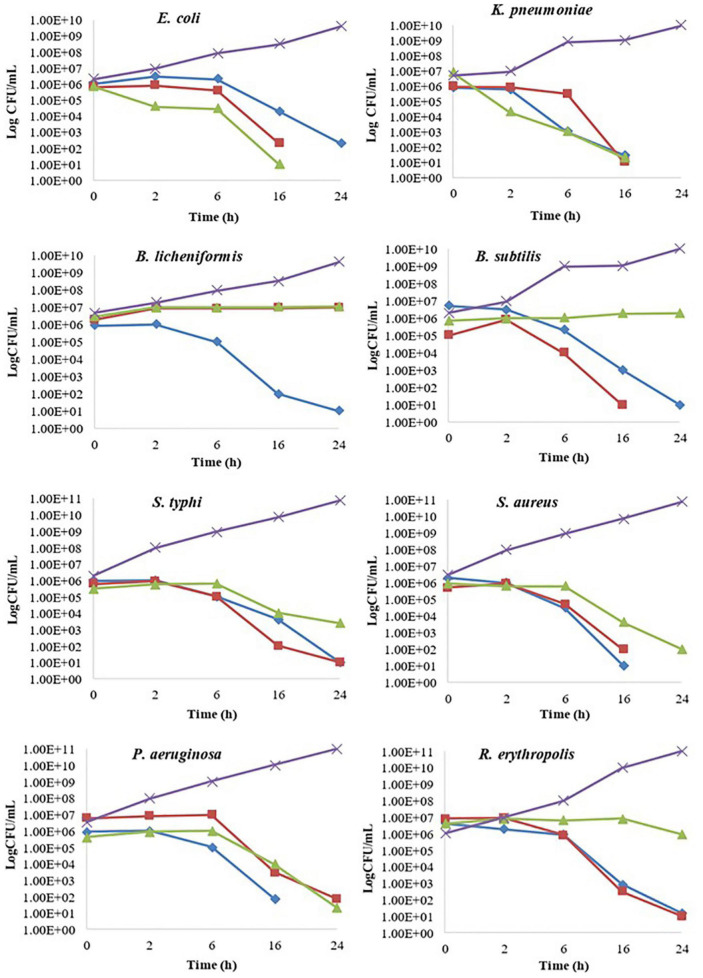
Kill time assay of *Berberis baluchistanica* bark, leaf, and root extracts against the target bacteria. In the graphs, ◆ represents bark ethanolic extract, ■ represents leaves ethanolic extract, ▲ represent roots ethanolic extract, and × refers to the positive control.

The MIC values of *B. baluchistanica* bark, leaves, and roots for different target test bacterial strains *B. subtilis*, *B. licheniformis*, *E. coli*, *K. pneumoniae*, *P. aeruginosa*, *R. erythropolis*, *S. typhi*, and *S. aureus* ranged from 1.56 to 25 mg ml^–1^ (Table 2). The MIC for the bark extract was 1.56 mg ml^–1^ against *S. aureus* and 3.12 mg ml^–1^ against *S. typhi*, while the bactericidal effect of the extract was above the MIC concentration range tested. For the leaf extract, the MIC was found as 1.56 mg ml^–1^ against *E. coli* and *S. aureus*, and the highest was 6.25 mg ml^–1^ against *R. erythropolis*. For the root extracts, the MIC was found to be 1.56 mg ml^–1^ against *E. coli* and *S. aureus* and highest against *K. pneumoniae* (25 mg ml^–1^). Based on the conducted research, it was found that the ethanolic extract of all parts strongly inhibited the growth of *E. coli*, *S. aureus*, *S. typhi*, and *P. aeruginosa*, while *K. pneumoniae* turned out to be the most resistant strains for root extracts (Figure 2). The MBC of the *B. baluchistanica* extracts were in the range of 3.12 to 25 mg ml^–1^. The root extracts for the bacterial strains *B. subtilis* and *R. erythropolis* were bacteriostatic, while both the leaf and root extracts were bacteriostatic for *B. licheniformis* (Table 2).

**TABLE 2:** Minimum inhibitory concentration and minimum bactericidal concentration of *Berberis baluchistanica*.

	Minimum inhibitory concentration			Minimum bactericidal concentration		
		
Microbial strain	Bark (mg/ml)	Leaves (mg/ml)	Root (mg/ml)	Bark (mg/ml)	Leaves (mg/ml)	Root (mg/ml)
*Bacillus subtilis*	3.12	3.12	6.25	6.25	6.25	Bacteriostatic
*Bacillus licheniformis*	6.25	6.25	12.5	12.5	Bacteriostatic	Bacteriostatic
*Escherichia coli*	3.12	1.56	1.56	6.25	3.12	3.12
*Klebsiella pneumoniae*	6.25	3.12	25	6.25	6.25	25
*Pseudomonas aeruginosa*	6.25	3.12	3.12	12.5	6.25	6.25
*Rhodococcus erythropolis*	6.25	6.25	6.25	12.5	12.5	Bacteriostatic
*Salmonella typhi*	3.12	6.25	3.12	3.12	12.5	6.25
*Staphylococcus aureus*	1.56	1.56	1.56	3.12	3.12	3.12

### Kill Time Assay

The kill time assay for target bacterial strains *B. subtilis*, *B. licheniformis*, *E. coli*, *K. pneumoniae*, *P. aeruginosa*, *R. erythropolis*, *S. typhi*, and *S. aureus* was performed by analyzing the growth decrease in log CFU/ml with time at different concentrations of *B. baluchistanica* bark, leaf, and root extracts compared to the positive controls, respectively. All extracts at their MIC inhibited the growth of bacteria and showed a significant (*p* < 0.05) decrease in bacterial viability over a period of 24 h compared to the positive control. The extract was considered bacteriostatic at the lowest concentration that reduced the original inoculum size to 0–3 log CFU/ml and bactericidal if the inoculum size was reduced by > 3 log CFU/ml. Almost all bacteria were sensitive against each extract and showed a bactericidal effect in 24 h of interaction. The effects of bark, leaf, and root extracts on each bacterial cell viability are shown in Figure 2. *B. licheniformis*, when treated with bark extract, showed a decrease in viability that was more than 3 log CFU/ml, indicating a bactericidal effect at 24 h. The leaf and root extracts showed a bacteriostatic effect against *B. licheniformis* as the bacterial count was less than 3 log CFU/ml at 24 h, whereas in the case of *B. subtilis*, when treated with bark and leaf extracts, bactericidal effects were observed after 6 h of incubation, and the decrease in bacterial count was more than 3 log CFU/ml. Complete bacterial elimination was observed after 16 h, but the root extracts showed a bacteriostatic effect.

### Antifungal Activity

**FIGURE 3:**
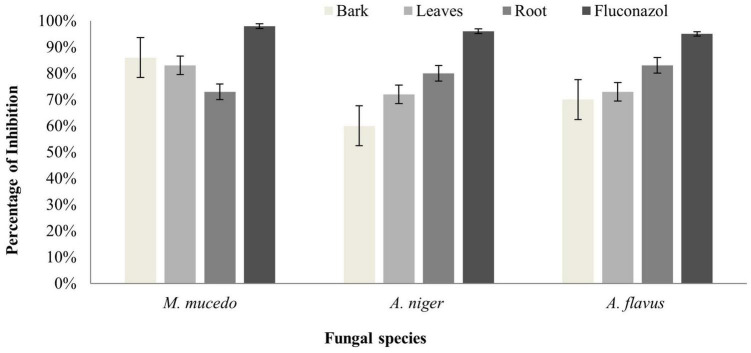
Antifungal activity of *Berberis baluchistanica* bark leaves and roots against fungal species *Mucor mucedo*, *Aspergillus niger*, and *Aspergillus flavus.* Bars represent the standard deviation of the mean.

The antifungal activity of *B. baluchistanica* bark leaves and root extracts was evaluated by measuring the percentage of growth inhibition potential against three filamentous fungi (*A. flavus*, *A. niger*, and *M. mucedo*). Comparing our results with the antifungal reference drug fluconazole, each extract was found active against all three fungi. The root extract possessed a potent antifungal activity against *A. flavus* with 83% growth inhibition and against *A. niger* with 80% but showed a relatively less effect on the growth of *M. mucedo* with 73% of inhibition. The bark extract was found active in a similar study against *M. mucedo* with 86% growth inhibition, followed by 70% against *A. flavus* and less inhibition against *A. niger* at 60%. The leaf extracts showed an active response of 83% inhibition against *M. mucedo*, followed by *A. flavus* and *A. niger* with 73 and 72% inhibition, respectively (Figure 3). The antifungal drug fluconazole was much potent with 98% inhibition against *M. mucedo*, 96% against *A. niger*, and 95% against *A. flavus* (Table 3).

**TABLE 3:** Antifungal activity of *Berberis baluchistanica* bark, leaves, and roots against three fungal species.

Plant parts	% inhibition *M. mucedo*	% inhibition *A. niger*	% inhibition *A. flavus*
Bark	86	60	70
Leaves	83	72	73
Roots	73	80	83
Fluconazole	98	96	95

*The results are expressed as percentage (%) of activity based on the potential of target fungi growth inhibition. Fluconazole = control.*

### Anti-leishmanial Assay

**FIGURE 4:**
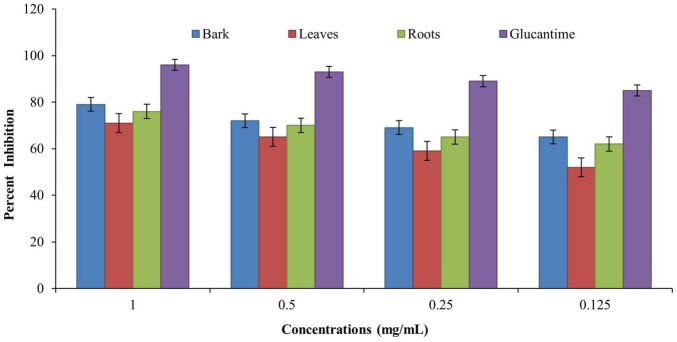
Anti-leishmanial activities of *Berberis baluchistanica* bark leaves and roots against promastigotes (*Leishmania major*). Bars represent the standard deviation of the mean.

Anti-leishmanial assay was used for determining the anti-leishmanial activity of promastigotes (*L. major*). A twofold serial dilution of each sample was carried out, and the activity was performed at different concentrations (1, 0.5, 0.25, and 0.125 mg/ml). The standard drug glucantime (IC_50_ = 5.41 ± 0.23 cmg/ml) was used to compare the parasite inhibition with each extract. The IC_50_ value was observed for each extract against *L. major*, and the bark showed good potential (IC_50_ = 4.95 ± 0.36 mg/ml), followed by the roots (IC_50_ = 7.07 ± 0.18 mg/ml) and leaves (IC_50_ = 8.25 ± 0.29 mg/ml) by comparing the values of each extract with the standards (Table 4). The results revealed that, in all concentrations, the standard drug glucantime reduced the viability compared with each extract (*P* < 0.05) and control (*P* < 0.05). The order of% inhibition activity was bark > roots > leaves. The highest activity was observed at the concentration of 1 mg/ml, and the viability increased with a decrease in concentration, as presented in Figure 4.

**TABLE 4:** Estimated IC_50_ values of bark, leaves, and roots of *Berberis baluchistanica* against promastigotes and HeLa cell line.

Samples	IC_50_ (mg/ml) ± SD Promastigotes	IC_50_ (mean ± SD, μg/ml) HeLa cell line
Bark	4.95 ± 0.36	12 ± 0.15
Leaves	8.25 ± 0.29	17 ± 0.21
Roots	7.07 ± 0.18	14 ± 0.15
Glucantime	5.41 ± 0.23	-
Doxorubicin	-	09 ± 0.12

*Glucantime and doxorubicin = control.*

### MTT Cell Assay

**FIGURE 5:**
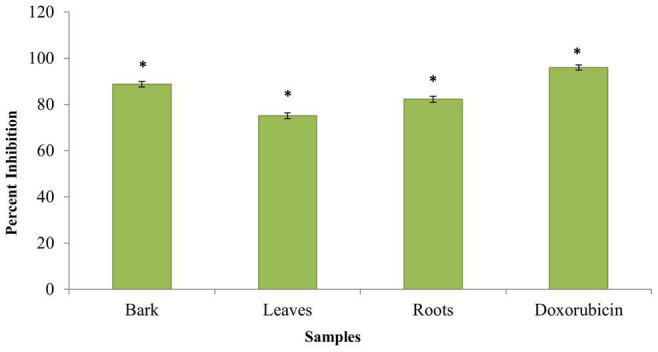
Anticancer activity of *Berberis baluchistanica* bark leaves and roots against HeLa cell line. Bars represent the standard deviation of the mean. Significant (*P* < 0.05) differences between groups are indicated by an asterisk.

Evaluation of the anticancer activity of the ethanolic extracts of the bark, leaves, and roots of the *B. baluchistanica* plant was done by using MTT cell assay against HeLa cell line. The assay was performed at 100 μg/ml for each extract, and doxorubicin was used as the standard drug to compare the inhibition with each extract. Each extract exhibited a higher anticancer (HeLa cell lines) activity and% inhibition in terms of IC_50_. Comparing the values to the standard (IC_50_ 09 ± 0.12 μg/ml), it was revealed that the ethanolic bark extract showed the highest anticancer activity with lowest IC_50_ (12 ± 0.15 μg/ml), followed by the roots (14 ± 0.15 μg/ml) and leaves (17 ± 0.21 μg/ml), respectively, as shown in Table 4. The results revealed that, in all concentrations, the standard drug doxorubicin reduced the viability compared with each extract, and the data was significantly different from each other (*P* < 0.05), as shown in Figure 5.

### Assessment of *in vitro* Anti-inflammatory Activity

#### Inhibition of Protein Denaturation

**FIGURE 6:**
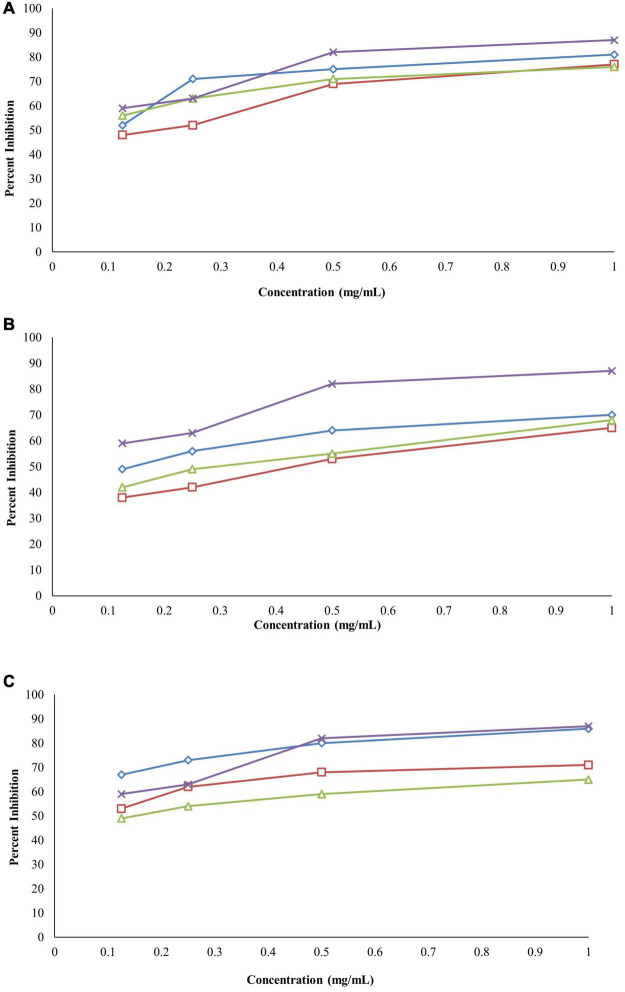
**(A)** Anti-inflammatory activity of *Berberis baluchistanica* bark leaves and roots by protein denaturation assay graphs. ◊ represents bark, □ represents leaves, △ represent roots, and × refers to the positive control (aspirin). **(B)** Anti-inflammatory activity of *B. baluchistanica* bark leaves and roots by proteinase inhibition assay. ◊ represents bark, □ represents leaves, △ represents roots, and × refers to the positive control (aspirin). **(C)** Anti-inflammatory activity of *B. baluchistanica* bark leaves and roots by heat-induced hemolysis assay. ◊ represents bark, □ represents leaves, △ represents roots, and × refers to the positive control (aspirin).

The ethanolic extracts of the bark, leaves, and roots were able to inhibit protein denaturation, and the inhibitions were in a concentration-dependent manner. The% inhibition of protein denaturation of all extracts was within the range from 48 to 81% at the concentration of 1–0.125 mg/ml, as shown in Figure 6A. The bark of *B. baluchistanica* revealed a significantly higher (*p* < 0.05) level of inhibition compared with those of the leaves and the roots. The IC_50_ value was observed for each extract, and the bark showed good potential (IC_50_ 0.64 ± 0.25 mg/ml), followed by the roots (IC_50_ = 0.67 ± 0.21 mg/ml) and the leaves (IC_50_ = 0.73 ± 0.13 mg/ml). By comparing the values of each extract to the standard, the order of% inhibition was bark > roots > leaves. The results revealed that, in all concentrations, the standard drug aspirin (IC_50_ = 0.43 ± 0.10 mg/ml) showed a relatively higher inhibition compared to the extracts (Table 5).

**TABLE 5:** IC_50_ value of *in vitro* anti-inflammatory assay carried out on *Berberis baluchistanica* bark, leaf, and root extracts.

Test samples	Concentration (mg/ml)	Protein inhibition denaturation IC_50_ (mg/ml)	Proteinase inhibition IC_50_ (mg/ml)	Heat-induced hemolysis IC_50_ (mg/ml)
Bark	1 0.5 0.25 0.125	0.64 ± 0.25	0.55 ± 0.12	0.48 ± 0.15
Leaves	1 0.5 0.25 0.125	0.73 ± 0.13	0.62 ± 0.23	0.52 ± 0.35
Roots	1 0.5 0.25 0.125	0.67 ± 0.21	0.69 ± 0.15	0.58 ± 0.05
Aspirin	1 0.5 0.25 0.125	0.43 ± 0.10	0.43 ± 0.10	0.43 ± 0.10

*Aspirin = control.*

#### Proteinase Inhibitory Activity

The bark, leaf, and root extracts of the *B. baluchistanica* plant were tested for its anti-inflammatory potential by proteinase inhibitory activity. The activity was performed at 1–0.125 mg/ml for each extract, and aspirin was used as the standard drug. Each extract exhibited good proteinase inhibition. The highest activity was observed at the concentration of 1 mg/ml, and the inhibition decreased with a decrease in concentration, as shown in Figure 6B. By comparing the obtained values of each extract to the standard drug aspirin (IC_50_ = 0.43 ± 0.10 mg/ml), it was revealed that ethanolic bark extract showed significantly higher (*p* < 0.05) levels of proteinase inhibition with lowest IC_50_ (0.55 ± 0.12 mg/ml), followed by the leaves (0.62 ± 0.23 mg/ml) and roots (0.69 ± 0.15 mg/ml), respectively. The order of% inhibition of extracts was bark > leaves > roots, as shown in Table 5.

#### Heat-Induced Hemolysis

The anti-inflammatory effect of the ethanolic extracts of the bark, leaves, and roots of the *B. baluchistanica* plant was carried out by heat-induced hemolysis. The percent inhibition of heat-induced hemolysis of red blood cells was analyzed at different concentrations (1–0.125 mg/ml). The ethanolic extracts of each part were able to inhibit hemolysis in a concentration-dependent manner, as presented in Figure 6C. The% inhibition of hemolysis was within the range from 49 to 86%. The bark extract showed significantly higher (*p* < 0.05) levels of hemolysis inhibition with the lowest IC_50_ value of 0.48 ± 0.15 mg/ml compared with the leaves (0.52 ± 0.35 mg/ml) and the roots (0.58 ± 0.05 mg/ml) of the plant. By comparing the% inhibition to the standard drug (aspirin, IC_50_ 0.43 ± 0.10 mg/ml), the order of the% inhibition of extracts was aspirin > bark > leaves > roots (Table 5).

## Discussion

The antibacterial activity of the *B. baluchistanica* bark, leaf, and root extracts was determined by using agar well diffusion method. All extracts showed effective antibacterial activity against the bacterial strains of *B. subtilis*, *B. licheniformis*, *E. coli*, *K. pneumonia*, *P. aeruginosa*, *R. erythropolis*, *S. typhi*, and *S. aureus*. The inhibition zone values for the tested agents are recorded in mm and presented in Table 1. The zones of inhibition less than 10 mm are referred to as weak; those with 10–16 mm are considered moderate, and those higher than 16 mm are active (Irshad et al., 2018). *S. aureus* was found to be the most sensitive strain in this study, with a maximum zone of inhibition of 23 ± 0.36 mm for the bark and 22 ± 0.12 mm for the leaves, followed by the root extracts (20.21 ± 0.06 mm). *E. coli* was found to be highly sensitive against root extracts with a maximum zone of inhibition at 23.14 ± 1.16 mm, at 22 ± 0.51 mm for the bark, and comparatively less sensitive to leave extracts at 19 ± 0.47 mm. The results revealed that all selected test pathogens were susceptible to each extract except the Gram-negative bacterium *K. pneumonia* that turned out to be the most resistant strain for the roots (7 ± 0.01 mm) and showed a minimum inhibition. In comparison with the standard drug doxycycline, the bark extract showed significant effectiveness against both Gram-positive and Gram-negative bacteria, followed by the leaf and root extracts (Figure 1). The results of the current study were in agreement with those previously reported (Uddin et al., 2021) and relatively higher than those found in the literature (Kakar et al., 2012). However, Pervez et al. (2019) evaluated the antibacterial potential of berberisinol that exhibited a significant activity against *E. coli*, *S. aureus*, and *S. pyogenes*, a moderate activity against *B. subtilis* and *K. pneumoniae*, and a weak activity against *P. aeruginosa*. The obtained results are in agreement with the recently reported antibacterial activity of the methanolic extract from the roots of *B. baluchistanica* against *S. aureus*, *B. subtilis*, *P. aeruginosa*, and *E. coli* with 30-, 22-, 19-, and 29-mm zones of inhibition (Idrees et al., 2021). A similar trend of interactions was observed with the previously documented results of other species of *Berberis* (Ilyas et al., 2021). The results suggested that each extract possesses a broad spectrum of antimicrobial potentials. The strong antibacterial effect of the obtained extracts is probably due to the presence of secondary metabolites, such as alkaloids, steroids, coumarin, saponins, and terpenoids, and a high content of phenolics and flavonoids which have been reported to be involved in the inhibition of nucleic acid biosynthesis and other metabolic processes (Iqbal et al., 2020).

The MIC values of the *B. baluchistanica* bark, leaves, and roots for different target bacterial strains *B. subtilis*, *B. licheniformis*, *E. coli*, *K. pneumoniae*, *P. aeruginosa*, *R. erythropolis*, *S. typhi*, and *S. aureus* ranged from 1.56 to 25 mg ml^–1^, and the MBC values of the *B. baluchistanica* extracts were in the range of 3.12 to 25 mg ml^–1^, respectively. The bark and leaf extracts showed the lowest MIC and MBC values compared with the root extract. The MIC values of the root extracts against multidrug resistant *E. coli* and *S. aureus* were found to be lower in the current study, while the MIC values for *K. pneumoniae* and *P. aeruginosa* were found to be relatively higher than previously reported (Kakar et al., 2012). The highest bacteriostatic effect of leaves was observed against *B. licheniformis*, and for root extracts it was observed against *B. licheniformis*, *B. subtilis*, and *R. erythropolis.* Other species of *Berberis* have also exhibited excellent antibacterial activities (Rafique and Salman, 2021). The hydro-alcoholic extracts of the stem and roots of *B. aristata*, *B. chitria*, and *B. lyceum* exhibited significant activity against *E. coli*, *K. pneumoniae*, *P. aeruginosa*, *S. aureus*, *S. pneumoniae*, and some other bacteria. However, none of the above-mentioned four *Berberis* species showed any activity against *S. typhimurium* except *B. lyceum* (Srivastava et al., 2006).

In the kill time assay, the target bacterial strains showed a decrease in viability when exposed to the extracts. During the first 2 h, there were an increased number of all the strains when treated with each sample. Later, there was a slower growth compared with the control, which clearly indicated that the extract of the bark, leaves, and roots inhibited the growth of the bacteria. The obtained results showed that the bark extract restrained the growth of *E. coli* cells. However, the leaf and root extracts, at a very minimal concentration, killed the cells by the end of 24 h. The effects of the bark, leaf, and root extracts on the viability of *K. pneumoniae* were somehow similar. The cells had a reduced target bacterial count with the passage of time, and this was completely killed at 24 h. *B. licheniformis*, when treated with the bark extract, showed a decrease in viability that was more than 3 log CFU/ml, indicating a bactericidal effect at 24 h. However, the leaf and root extracts showed a bacteriostatic effect as the bacterial count was less than 3 log CFU/ml at 24 h, whereas in the case of *B. subtilis*, when treated with the bark and leaf extracts, bactericidal effects were observed after 6 h of incubation, and the decrease in bacterial count was more than 3 log CFU/ml. Complete bacterial elimination was observed after 16 h; however, the root extracts showed a bacteriostatic effect. Complete elimination of *P. aeruginosa*, *R. erythropolis*, *S. typhi*, and *S. aureus* cells was observed after 24 h of treatment with the bark, leaf, and root extracts. The bacterial growth in each control sample was increased with the passage of time. The obtained results supported previous research that reported a complete reduction in the growth of *S. typhi*, *E. coli*, and *S. aureus* in the kill time assay (Sadiq et al., 2017; Akbar et al., 2019b).

The antifungal activity of the *B. baluchistanica* bark, leaf, and root extracts was evaluated by measuring the percentage of inhibition zone against three filamentous fungi (*A. flavus*, *A. niger*, *M. mucedo*). These fungal pathogens were cultured using autoclaved potato dextrose agar. Comparing our results with the antifungal drug fluconazole, each extract was found to be active against all three fungi. The root extract possessed potent antifungal activity against *A. flavus* with 83% of growth inhibition and against *A. niger* with 80% but showed a relatively less effect on the growth of *M. mucedo* with 73% of inhibition zone. The bark extract was found to be active in a similar study against *M. mucedo* with 86% of inhibition, followed by 70% against *A. flavus* and less inhibition against *A. niger* at 60%. The leaf extract showed an active response of 83% inhibition against *M. mucedo*, followed by *A. flavus* and *A. niger* with 73 and 72% inhibition, respectively. The antifungal drug fluconazole was much potent with 98% inhibition against *M. mucedo*, 96% against *A. niger*, and 95% against *A. flavus* (Table 3). Recently, Uddin et al. (2021) reported the BBS-NiONP antifungal response against three fungal species: *A. alternate*, *A. niger*, and *F. oxysporum* with the following manner of inhibition: *A. alternata* > *A. niger* > *F. oxysporum*. Previously, Pervez et al. (2019) determined the antifungal potential of the compound berberisinol against the different fungal strains of *C. glabrata*, *A. flavus*, *M. canis*, *F. solani*, *C. albicans*, and *A. niger* that showed no significant antifungal activity against all the fungal strains.

According to Khan et al. (2012), the crude methanolic extract of the roots of *B. baluchistanica* showed a maximum activity against *C. albicans* and *M. canis* with% inhibition of 62 and 51%, respectively, and least inhibition (11%) against *A. flavus.*

Leishmaniasis is an infectious parasitic disease that is caused by a *Leishmania* strain that causes cutaneous, mucocutaneous, and visceral diseases, especially in developing countries. The disease has not been controlled due to the absence of an effective and easily available low-cost treatment. *B. baluchistanica* might control the disease due to its bioactive components and strong antimicrobial potentials.

Anti-leishmanial assay was the method used for determining the leishmanicidal activity, and promastigote (*L. major*) species was used for investigation. A twofold serial dilution of each sample was carried out, and the activity was performed at different concentrations (1, 0.5, 0.25, and 0.125 mg/ml). The standard drug glucantime (IC_50_ 5.41 ± 0.23 mg/ml) was used to compare the parasite inhibition with each extract. The activity was carried out under an incubation period of 48 h at 22°C. Moreover, 50% inhibitory concentration was observed for each extract against *L. major*, and the bark showed good potential (IC_50_ 4.95 ± 0.36 mg/ml), followed by the roots (IC_50_ 7.07 ± 0.18 mg/ml) and the leaves (IC_50_ 8.25 ± 0.29 mg/ml), as derived by comparing the values of each extract to the standards. The order of% inhibition was bark > roots > leaves. The results revealed that, in all the concentrations, the standard drug glucantime reduced the viability compared with each extract (*P* < 0.05) and control (*P* < 0.05). The highest activity was observed in the concentration of 1 mg/ml, and the viability increased with a decrease in concentration. The obtained results are in agreement with recently documented data of other species of *Berberis* (Najim and Hassan, 2020). Previously, Baloch et al. (2013) reported the strong anti-leishmanial potential of *B. baluchistanica* roots.

Cancer is a disease that is explained by cells in the human body that are increasing but with failure to be controlled. About 60% of medications allowed for cancer treatment are of a natural source. The anticancer activity was not reported and published by other researchers to date, but different species of genus *Berberis* are reported to have *in vitro* cytotoxic activity against different destructive cell lines (El Fakir et al., 2021; Ilyas et al., 2021). An evaluation of the anticancer activity of the ethanolic extracts of the bark, leaves, and roots of the *B. baluchistanica* plant was done by using MTT cell assay against HeLa cell line. The assay was performed at 100 μg/ml for each extract, and doxorubicin was used as the standard drug with which to compare the inhibition of each extract. Comparing the values to the standard (IC_50_ = 09 ± 0.12 μg/ml), it was revealed that the ethanolic bark extract showed the highest anticancer activity with the lowest IC_50_ (12 ± 0.15 μg/ml), followed by the roots (14 ± 0.15 μg/ml) and the leaves (17 ± 0.21 μg/ml), respectively. Each extract exhibited a higher anticancer (HeLa cell lines) activity, and the% inhibitions are shown in Table 4 and Figure 5.

### Assessment of *in vitro* Anti-inflammatory Activity

Inflammation is the response of living tissues as a result of any damage or injury. It is exemplified by pain, fever, redness, and loss of body physiological functions. Different steroidal anti-inflammatory drugs and non-steroidal anti-inflammatory drugs are being used to treat the effects of prolonged and severe inflammation, but the long-term use of these drugs may also cause adverse effects. Therefore, safe and long-term-use anti-inflammatory agents need to be discovered, and plant-derived medicines are perceived to be efficient, of low cost, and with less adverse effects. The different parts of the medicinal plant *B. baluchistanica* were evaluated for anti-inflammatory activity by three methods, including protein denaturation, proteinase inhibition, and heat-induced hemolysis.

Denaturation of tissue proteins is one of the main causes of inflammatory diseases. The production of auto-antigens in different inflammatory diseases may be due to protein denaturation. Chemical agents that have the potential to prevent protein denaturation would be valuable for the development of anti-inflammatory drugs (Chandra et al., 2012). Ethanolic extracts of the bark, leaves, and roots were able to inhibit protein denaturation, and the inhibitions were in a concentration-dependent manner. The% inhibition of each extract at different concentrations (1–0.125 mg/ml) on protein denaturation is shown in Figure 6A. The bark extract of *B. baluchistanica* revealed a significantly higher (*p* < 0.05) level of inhibition compared with the leaves and the roots. The IC_50_ was observed for each extract, and the bark showed good potential (IC_50_ = 0.64 ± 0.25 mg/ml), followed by the roots (IC_50_ = 0.67 ± 0.21 mg/ml) and the leaves (IC_50_ = 0.73 ± 0.13 mg/ml). By comparing the values of each extract to the standard, the order of% inhibition was bark > roots > leaves. The results revealed that, in all the concentrations, the standard drug aspirin showed a relatively higher inhibition compared with each extract.

The bark, leaf, and root extracts of the *B. baluchistanica* plant were tested for its anti-inflammatory potential by proteinase inhibitory activity. Each extract exhibited high proteinase inhibition, and the results are shown in Figure 6B. The% inhibitions were within the range of 38–70%. The bark extract has shown a significantly higher (*p* < 0.05) proteinase inhibition compared with the leaves and the roots. The order of% inhibition of proteinase activity was bark > leaves > roots. The activity was performed at 1–0.125 mg/ml for each extract, and aspirin was used as the standard drug to compare the inhibition with each extract. Comparing the values to the standard, it was revealed that the ethanolic bark extract showed the highest proteinase inhibition with the lowest IC_50_ (0.55 ± 0.12 mg/ml), followed by the leaves (0.62 ± 0.23 mg/ml) and the roots (0.69 ± 0.15 mg/ml), respectively. The highest activity was observed in the concentration of 1 mg/ml, and the inhibition decreased with a decrease in concentration.

The anti-inflammatory effect of the ethanolic extracts of the bark, leaves, and roots of *B. baluchistanica* plant was carried out by heat-induced hemolysis. The percent inhibition of heat-induced hemolysis of red blood cells was analyzed at different concentrations of 1–0.125 mg/ml. The ethanolic extracts of each part were able to inhibit hemolysis in a concentration-dependent manner. The% inhibition of hemolysis was within the range from 49 to 86%. The bark extract showed significantly higher (*p* < 0.05) levels of hemolysis inhibition with the lowest IC_50_ value (0.48 ± 0.15 mg/ml) compared with the leaves (0.52 ± 0.35 mg/ml) and the roots (0.58 ± 0.05 mg/ml) of the plant. By comparing the% inhibition with the standard drug aspirin with IC_50_ (0.43 ± 0.10 mg/ml), the order of the% inhibition of extracts was aspirin > bark > leaves > roots. Previously, Siddiqui et al. (2017) evaluated the anti-inflammatory property of the fruit of *B. baluchistanica* on mice by using acetic acid-induced writhing method and formalin method and claimed that the fruit could be used as a good anti-inflammatory drug. The obtained results are in agreement with the recently reported anti-inflammatory effect of *B. baluchistanica* by Pervez et al. (2020) in acetic acid-induced writhing and formalin-induced flinching behavior tests. Phytochemicals such as phenolics, flavonoids, saponins, and tannins are able to protect the protein membranes from denaturation by binding the cat ions and other molecules. According to Abate et al. (2021), the higher the concentration of these classes of compounds, the lower the inhibition concentration IC_50_ needed to reduce the inflammation response. Therefore, polyphenolic compounds present in ethanolic extracts of bark leaves and roots could be the possible reason of the stabilization of the lysosomal membrane by its anti-denaturation property.

## Conclusion

In the perspective of the research conducted, it can be revealed that the *B. baluchistanica* bark, leaf, and root extracts were found to have crucial antibacterial, antifungal, anti-inflammatory, anti-leishmanial, and anticancer potentials. From the results, it can be concluded that the *B. baluchistanica* bark, leaf, and root extracts could be good sources of effective drugs for various infections against humans. Each part of the plant was found to be potentially active for antimicrobial and other activities, whereas the bark was found to be relatively more potent than the other parts in some biological activities. However, more investigations based on these preliminary studies are needed to explore the bioactive molecules along with the mechanisms of action which are responsible for their effectiveness.

## Data Availability Statement

The raw data supporting the conclusions of this article will be made available by the authors, without undue reservation.

## Author Contributions

ZG collected the data and analyzed and drafted the manuscript. AA and SL conceptualized, designed, and supervised the study. AA reviewed the draft and finalized it. All authors reviewed the final manuscript.

## Conflict of Interest

The authors declare that the research was conducted in the absence of any commercial or financial relationships that could be construed as a potential conflict of interest.

## Publisher’s Note

All claims expressed in this article are solely those of the authors and do not necessarily represent those of their affiliated organizations, or those of the publisher, the editors and the reviewers. Any product that may be evaluated in this article, or claim that may be made by its manufacturer, is not guaranteed or endorsed by the publisher.
